# Differences in ventricular wall composition may explain inter-patient variability in the ECG response to variations in serum potassium and calcium

**DOI:** 10.3389/fphys.2023.1060919

**Published:** 2023-10-11

**Authors:** Hassaan A. Bukhari, Carlos Sánchez, Pablo Laguna, Mark Potse, Esther Pueyo

**Affiliations:** ^1^ BSICoS Group, I3A Institute, University of Zaragoza, IIS Aragón, Zaragoza, Spain; ^2^ CIBER en Bioingeniería, Biomateriales y Nanomedicina (CIBER-BBN), Zaragoza, Spain; ^3^ Carmen Team, Inria Bordeaux—Sud-Ouest, Talence, France; ^4^ University of Bordeaux, IMB, UMR 5251, Talence, France; ^5^ IHU Liryc, Electrophysiology and Heart Modeling Institute, Fondation Bordeaux Université, Bordeaux, France

**Keywords:** ECG, T wave morphology, QRS complex morphology, transmural heterogeneity, potassium, calcium, hemodialysis, heart-torso models

## Abstract

**Objective:** Chronic kidney disease patients have a decreased ability to maintain normal electrolyte concentrations in their blood, which increases the risk for ventricular arrhythmias and sudden cardiac death. Non-invasive monitoring of serum potassium and calcium concentration, [K^+^] and [Ca^2+^], can help to prevent arrhythmias in these patients. Electrocardiogram (ECG) markers that significantly correlate with [K^+^] and [Ca^2+^] have been proposed, but these relations are highly variable between patients. We hypothesized that inter-individual differences in cell type distribution across the ventricular wall can help to explain this variability.

**Methods:** A population of human heart-torso models were built with different proportions of endocardial, midmyocardial and epicardial cells. Propagation of ventricular electrical activity was described by a reaction-diffusion model, with modified Ten Tusscher-Panfilov dynamics. [K^+^] and [Ca^2+^] were varied individually and in combination. Twelve-lead ECGs were simulated and the width, amplitude and morphological variability of T waves and QRS complexes were quantified. Results were compared to measurements from 29 end-stage renal disease (ESRD) patients undergoing hemodialysis (HD).

**Results:** Both simulations and patients data showed that most of the analyzed T wave and QRS complex markers correlated strongly with [K^+^] (absolute median Pearson correlation coefficients, *r*, ranging from 0.68 to 0.98) and [Ca^2+^] (ranging from 0.70 to 0.98). The same sign and similar magnitude of median *r* was observed in the simulations and the patients. Different cell type distributions in the ventricular wall led to variability in ECG markers that was accentuated at high [K^+^] and low [Ca^2+^], in agreement with the larger variability between patients measured at the onset of HD. The simulated ECG variability explained part of the measured inter-patient variability.

**Conclusion:** Changes in ECG markers were similarly related to [K^+^] and [Ca^2+^] variations in our models and in the ESRD patients. The high inter-patient ECG variability may be explained by variations in cell type distribution across the ventricular wall, with high sensitivity to variations in the proportion of epicardial cells.

**Significance:** Differences in ventricular wall composition help to explain inter-patient variability in ECG response to [K^+^] and [Ca^2+^]. This finding can be used to improve serum electrolyte monitoring in ESRD patients.

## 1 Introduction

An estimated 13.4% of the world population is affected by chronic kidney disease (Lv and Zhang, 2019). All stages of this disease, but particularly end-stage renal disease (ESRD), are associated with life-threatening arrhythmias, which increase mortality and decrease quality of life (Soar et al., 2010; Levis, 2012; Hill et al., 2016; Weiss et al., 2017; Noordam et al., 2019). Five to seven million ESRD patients require renal replacement therapy worldwide and many of them die due to lack of access to affordable treatment (Lv and Zhang, 2019). Abnormal levels of serum potassium ([K^+^]) and calcium ([Ca^2+^]), in the form of hypo- or hyperkalemia and hypo- or hypercalcemia (Soar et al., 2010; Levis, 2012; Weiss et al., 2017; Noordam et al., 2019), increase the risk of ventricular arrhythmias and sudden cardiac death in ESRD patients (Bozbas et al., 2007; Weiss et al., 2017).

The electrocardiogram (ECG) is known to be affected by [K^+^] and [Ca^2+^] variations (Lanari et al., 1964; Van Mieghem et al., 2004; El-Sherif and Turitto, 2011; Noordam et al., 2019). On this basis, ECG-derived estimators of [K^+^] and [Ca^2+^] have been proposed in several studies. Some of these estimators are based on the QRS complex duration, the QT interval and the T wave amplitude, slope or slope-to-amplitude ratio (Parham et al., 2006; Severi et al., 2009b; Corsi et al., 2012; Astan et al., 2015; Dillon et al., 2015; Attia et al., 2016; Corsi et al., 2017; Weiss et al., 2017; Yoon et al., 2018; Noordam et al., 2019; Pilia et al., 2020). Other studies have investigated model-based markers of T wave morphology (Rodrigues et al., 2020), sympathetic activity-related T wave instability during HD (Schüttler et al., 2021) and descriptors of the sine wave shape, amplitude and time voltage area of the QRS complex (Ojanen et al., 1999; An et al., 2012; Curione et al., 2013; Pilia et al., 2020). To characterize overall variations in the morphology of the QRS complex and the T wave, in previous studies we have developed [K^+^] and [Ca^2+^] estimators using non-linear dynamics and time-warping techniques and we have evaluated them during and after HD in ESRD patients (Bukhari et al., 2019; Srinivasan et al., 2020; Palmieri et al., 2021a; Palmieri et al., 2021b; Bukhari et al., 2021; Bukhari et al., 2022a; Bukhari et al., 2022b).

A common weakness of all the proposed markers is that their relationships with electrolyte concentrations vary strongly between patients (Buemi et al., 2005; Montague et al., 2008; Alabd et al., 2011; Green et al., 2013; Pilia et al., 2020; Palmieri et al., 2022; Palmieri et al., 2021b). The cause of this variability is presently unknown. Here, we hypothesized that inter-individual differences in cell type distribution across the ventricular wall could help to explain such variability between patients. We tested this hypothesis using a population of realistic computational models, based on the anatomy of a real subject. We characterized ECG features, including several proposed markers for [K^+^] and [Ca^2+^], in models with different proportions of endocardial, midmyocardial and epicardial myocytes at varying [K^+^] and [Ca^2+^], and compared the results to measurements in 29 ESRD patients.

We found that the relationships between electrolyte concentrations in the models were similar to those in patients and the variability in the relationships could indeed be explained, partly, by differences in ventricular wall composition.

## 2 Materials and methods

### 2.1 *In silico* population of human heart-torso models

**FIGURE 1:**
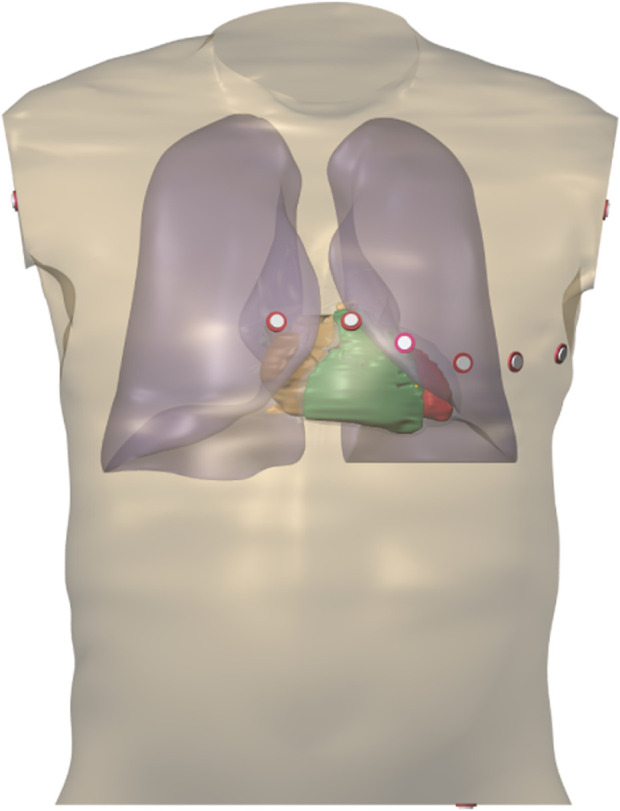
3D heart-torso model used for ECG simulations.

A population of coupled whole-ventricle and torso models was built taking as a basis the computed tomography (CT) data of a patient (Kania et al., 2017), as shown in Figure 1. The model included the ventricular wall, ventricular and atrial cavities, torso surface, lungs and an approximate anisotropic skeletal muscle layer, which were segmented from the CT data. A hexahedral mesh of the heart with 200 μm resolution and a torso mesh with 1 mm resolution were created.

Ventricular electrical propagation was simulated with a monodomain reaction-diffusion model:

$$\frac{\partial V_{\text{m}}}{\partial t}=\frac{1}{C_{\text{m}}}\left(\beta^{-1}\nabla \cdot \left(G\nabla V_{\text{m}}\right)-I_{\text{ion}}\right),$$ (1)

where *V*_m_ is the transmembrane potential, *C*_m_ the membrane capacitance, *β* the membrane surface-to-volume ratio (the amount of membrane found in a given volume of tissue), *I*_ion_ the sum of all transmembrane ionic currents, and *G* the monodomain conductivity tensor *G*_int_ *G*_ext_/(*G*_int_ + *G*_ext_) with *G*_int_ and *G*_ext_ representing the intracellular and extracellular conductivity tensor fields, respectively. The fiber orientations used to compute the conductivity tensors were assigned with a rule-based method (Streeter et al., 1969; Potse et al., 2006). *β* was set to 800 cm^−1^ for myocardium (Potse, 2018). The *G*_int_ and *G*_ext_ tensors were defined according to the following values: intracellular transverse and longitudinal conductivities were 0.3 and 3 mS/cm and extracellular transverse and longitudinal conductivities were 1.2 and 3 mS/cm, respectively (Potse, 2018).

Temporal integration was done with a forward Euler scheme with a time step of 0.01 ms and a spatial resolution of 0.02 cm. For accuracy, gating variables in the membrane model were integrated with the Rush-Larsen method (Rush and Larsen, 1978), with a time step of 0.01 ms. The simulations were performed using a recent version of the Propag-5 software (Krause et al., 2012) at a stimulation frequency of 1 Hz.

Cellular electrophysiology was represented by the human ventricular myocyte model of Ten Tusscher and Panfilov (2006). The updates to the Ten Tusscher-Panfilov model published by Severi et al. (2009a) were incorporated to adequately represent the relationship between AP duration (APD) and [Ca^2+^]. The L-type calcium current was described as:

$$\begin{array}{ll} I_{\text{CaL}} & =G_{\text{CaL}}\;d\;f\;f_{2}\;f_{\text{Cass}}\;4\;\frac{\left(V_{\text{m}}-15\right)F^{2}}{RT}\\ & \;\times \frac{\left[{Ca}^{2+}\right]-0.25\;{\left[{Ca}^{2+}\right]}_{\text{ss}}\;e^{2\left(V_{\text{m}}-15\right)F/\left(RT\right)}}{1-e^{2\left(V_{\text{m}}-15\right)F/\left(RT\right)}}, \end{array}$$ (2)

where *G*_CaL_ is the maximal *I*_CaL_ conductance, *d* is a voltage-dependent activation gate, *f* and *f*_2_ are voltage-dependent inactivation gates, *f*_Cass_ is an inactivation gate dependent on the calcium concentration in the dyadic space (in mM), denoted by ${\left[{Ca}^{2+}\right]}_{\text{ss}}$ , *F* is the Faraday constant, *R* is the gas constant, *T* is the temperature and *V*_m_ is the transmembrane potential (in mV). The updated formulations for *f*_2_ and *f*_Cass_ of Severi et al. (2009a) read

$$\frac{df_{\text{Cass}}}{dt}=k\frac{f_{\text{Cass,inf}}-f_{\text{Cass}}}{\tau_{f_{\text{Cass}}}},$$ (3)

where *k* = 0 if *f*_Cass,inf_ > *f*_Cass_ and *V* > − 60 mV, and *k* = 1 otherwise,

$$f_{\text{Cass,inf}}=\frac{0.9}{1+\exp\left(\frac{{\left[{Ca}^{2+}\right]}_{\text{ss}}-1.95}{0.15}\right)}+0.1,$$ (4)

$$\tau_{f_{\text{Cass}}}=\frac{80}{1+{\left(\frac{{\left[{Ca}^{2+}\right]}_{\text{ss}}}{0.05}\right)}^{2}}+1,$$ (5)

and

$$f_{2,inf}=\frac{0.3}{1+e^{\left(\frac{V_{\text{m}}+35}{7}\right)}}+0.7,$$ (6)

$$\begin{array}{ll} \tau_{f_{2}} & =600\;\exp\left(-\frac{{\left(V_{\text{m}}+25\right)}^{2}}{170}\right)+\frac{31}{1+\exp\left(\frac{25-V_{\text{m}}}{10}\right)}\\ & \;+\frac{16}{1+\exp\left(\frac{V_{\text{m}}+30}{10}\right)}. \end{array}$$ (7)

The initial state for each simulation was pre-calculated from a single cell simulation, one for each cell type: endocardial, midmyocardial (M cells) and epicardial. The values of the model state variables after 1000 paced beats were considered as representative of the cell at steady state.

A total of 7 whole-ventricle models with variations in the proportions of endocardial, midmyocardial and epicardial cells were simulated, with the thickness of endocardial and midmyocardial layers ranging from 10% to 50% and the epicardial layer from 20% to 60%. The different cell types differ in the ion channel characteristics, as described in Ten Tusscher et al. (2004) and Ten Tusscher and Panfilov (2006). We used the notation C*uvw*, where C stands for the word “case” and *u*, *v* and *w* denote the thicknesses of the endocardial, midmyocardial, and epicardial layers in tenths of the total wall thickness, respectively (e.g., *C*532 represents the case with 50%, 30%, and 20% of endocardial, midmyocardial and epicardial cells, respectively) (Bukhari et al., 2021). The population of ventricular models included the following combinations of transmural heterogeneities: *C*136, *C*154, *C*316, *C*334, *C*352, *C*514, *C*532.

### 2.2 ECG simulation and processing

The extracellular potential, *ϕ*_ext_, was computed by solving

$$\nabla \cdot \left(\left(G_{\text{int}}+G_{\text{ext}}\right)\nabla \phi_{\text{ext}}\right)=-\nabla \cdot \left(G_{\text{int}}\nabla V_{\text{m}}\right)$$ (8)

in the torso model, with *V*_m_ simulated by the monodomain reaction-diffusion model (1) (Potse, 2018). Since we needed to know *ϕ*_ext_ only at a few locations for the computation of the ECG we used a Green’s function of the operator ∇ ⋅ ((*G*_int_ + *G*_ext_)∇.) for each of these locations to solve this equation efficiently. The ECG leads can be represented by a linear combination of Green’s functions because each recorded ECG lead is a linear combination of *ϕ*_ext_ at two or more points. These linear combinations of Green’s functions are termed lead fields (McFee and Johnston, 1954; Colli-Franzone et al., 2000). Our lead fields were computed by solving an equation similar to (8) but as a lead field needs to be computed only once for each ECG lead this approach is much more efficient than solving (8) for each time step in each simulation (Potse, 2018). Five-beat ECGs were simulated at a sampling frequency of 1 kHz.

Principal component (PC) analysis was spatially applied to the T waves (or QRS complexes) of the eight independent leads (Castells et al., 2007) to enhance the T wave (or QRS complex) energy. For each model in the population, the coefficients defining the PC transformation were obtained from the eigenvectors of the 8 × 8 inter-lead auto-correlation matrix estimated by including all segmented T waves (or QRS complexes) at the physiological values of [K^+^], i.e., 5.4 mM, and [Ca^2+^], i.e., 2 mM. Individual ECG recordings of the same model corresponding to other values of [K^+^] and/or [Ca^2+^] were then projected onto the direction of the first PC determined as described above and used for further analysis. We chose to analyze the transformed lead, which represented the main characteristics from the combination of all leads and allowed for an improved morphological characterization.

A wavelet-based single-lead delineation method was used for QRS detection and wave delineation of each of the 12 leads (Martínez et al., 2004). Similarly, the T waves and QRS complexes in the first PC were delineated using the single-lead delineation algorithm described by Martínez et al. (2004). The onset, peak and end of the T waves and QRS complexes were determined and used for subsequent computation of T wave and QRS complex markers.

Simulation results were compared with the results obtained by applying the same analysis to ECG recordings from patients. The study population included 29 ESRD patients from Hospital Clínico Universitario de Zaragoza, Spain, from which 48-h 12-lead ECGs were acquired (see Supplementary Table S1 in the Supplementary Material with [K^+^], [Ca^2+^] and RR values in the patients). T waves (QRS complexes, respectively) in the first PC were obtained and delineated to compute the below described markers (Palmieri et al., 2021a; Bukhari et al., 2022a; Bukhari et al., 2022b).

### 2.3 Duration, amplitude and morphology-based ECG markers

#### 2.3.1 Duration- and amplitude-based ECG markers

**FIGURE 2:**
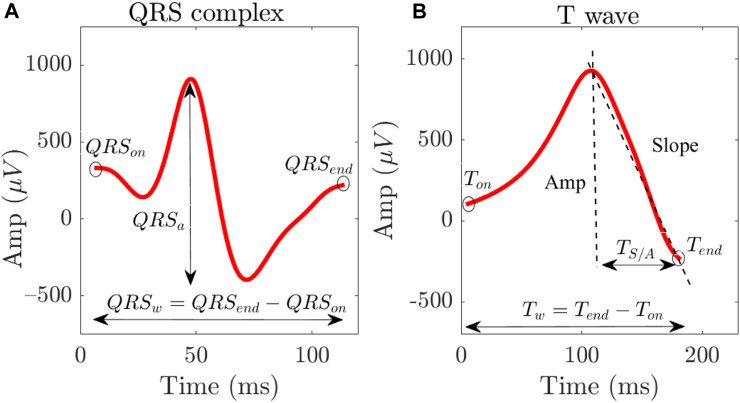
QRS complex **(A)** and T wave **(B)** from a particular simulated case corresponding to a 3D torso-heart model, with the computed duration and amplitude-based ECG markers.

Time- and amplitude-based T wave and QRS complex markers were computed from the last T wave and QRS complex of each simulated ECG projected onto the first PC. The analyzed T wave and QRS complex markers as shown in Figure 2, computed at varying [K^+^], [Ca^2+^] and their combinations, included.

• *T*_w_, representing T wave width calculated from T wave onset to T wave end (expressed in ms),
• *T*_S/A_, representing the ratio between the maximal downward slope (in absolute value) and the amplitude of the T wave (expressed in 1/ms) (Corsi et al., 2012; Corsi et al., 2017),
• *QRS*_w_, representing QRS complex width calculated from QRS complex onset to QRS complex end (expressed in ms), and
• *QRS*_a_, representing QRS amplitude calculated from minimum to maximum amplitude of QRS complex (expressed in mV).

#### 2.3.2 Morphological variability-based ECG markers

Morphology-based T wave and QRS complex markers were computed using the time-warping methodology described previously (Ramírez et al., 2017; Bukhari et al., 2022a). For each model in the population, reference T waves and QRS complexes were calculated from the last beat of the PC-transformed ECG at minimum [K^+^] (3 mM) and maximum [Ca^2+^] (3.2 mM). We considered the reference for the simulated T wave (or QRS complex) in the same way as considered for the ESRD patients so as to perform the comparison on a similar basis. In the patients, the reference was computed at the end of the HD session when [K^+^] was minimum, closer to 3 mM, and [Ca^2+^] was maximum, closer to 3.2 mM, as this is the time when the patient was discharged from hospital with restored serum ion levels, thus being an acceptable reference for ambulatory monitoring (Bukhari et al., 2021; Bukhari et al., 2022a; Bukhari et al., 2022b).

**FIGURE 3:**
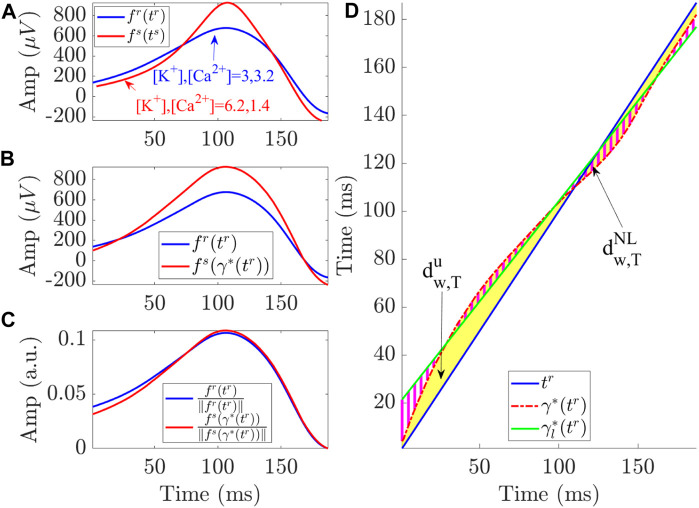
Linear and non-linear time warping for a particular simulated case (*C*154) corresponding to a 3D torso-heart model. **(A)** shows the reference (blue) and investigated (red) T waves obtained for the indicated [K^+^] and [Ca^2+^] values. **(B)** shows the warped T waves, which have the same duration while keeping the original amplitude. **(C)** depicts the warped T waves after normalization by their L2-norms. The area (yellow region) between both T waves in **(D)** represents $d_{w,\text{T}}^{\text{u}}$ , which quantifies the total amount of warping. The green solid line is the linear regression function $\gamma_{l}^{*}\left(t^{r}\right)$ best fitted to the optimal warping function, γ*(*t* ^*r*^), with the latter being represented by the red dotted line. The marker $d_{w,\text{T}}^{\text{NL}}$ quantifies the non-linear warping by computing the area of the dashed magenta region between γ*(*t* ^*r*^) and $\gamma_{l}^{*}\left(t^{r}\right)$ .

The simulated T wave (or QRS complex) at a particular electrolyte level was expressed as **f** ^*s*^(**t** ^*s*^) and the reference simulated T wave (or QRS complex) as **f** ^*r*^(**t** ^*r*^). Figure 3A shows **f** ^*r*^ and **f** ^*s*^, with their respective time domains, **t** ^*r*^ and **t** ^*s*^. Let *γ*(**t** ^*r*^) be the warping function that relates **t** ^*r*^ and **t** ^*s*^, such that **f** ^*s*^(*γ*(**t** ^*r*^)) denotes the warping of **f** ^*s*^(**t** ^*s*^) using *γ*(**t** ^*r*^). A dynamic programming algorithm was used to obtain the function γ*(**t** ^*r*^) that optimally warps **f** ^*r*^(**t** ^*r*^) into **f** ^*s*^(**t** ^*s*^). This function is shown in Figure 3D. The warped T wave, **f** ^*s*^(γ*(**t** ^*r*^)), is shown in Figure 3B, together with the reference T wave, **f** ^*r*^(**t** ^*r*^). Figure 3C represents normalized reference and warped T wave.

The marker $d_{w,\text{T}}^{\text{u}}$ , shown in Figure 3D, was used to quantify the level of warping required to optimally align the T waves **f** ^*s*^(**t** ^*s*^) and **f** ^*r*^(**t** ^*r*^) as computed in previous studies (Ramírez et al., 2017; Palmieri et al., 2021a; Bukhari et al., 2021). The superscript *u* indicates unsigned, meaning that the computation was based on the absolute value of the differences between the elements in vector γ*(**t** ^*r*^) and in vector **t** ^*r*^, thus not accounting for the sign of such differences. The amplitude marker *d*_a,T_ was computed from the area contained between **f** ^*r*^(**t** ^*r*^) and **f** ^*s*^(γ*(**t** ^*r*^)) normalized by the L2-norm of **f** ^*r*^(**t** ^*r*^), thus quantifying amplitude differences after time warping the two T waves (Ramírez et al., 2017; Palmieri et al., 2021a; Bukhari et al., 2021). Similarly, $d_{\text{w},\text{Q}}^{\text{u}}$ and *d*_a,Q_ were quantified from QRS complexes as can be seen from Supplementary Figure S1 in the Supplementary Material and described in Bukhari et al. (2022a).

The markers $d_{w,\text{T}}^{\text{u}}$ and *d*_a,T_ incorporate information from the linear and non-linear warping required to fit the two T waves in the time and amplitude domain. The markers $d_{w,\text{T}}^{\text{NL}}$ and $d_{a,\text{T}}^{\text{NL}}$ were calculated to restrictively quantify the non-linear part of the warping (Ramírez et al., 2017; Palmieri et al., 2021a; Bukhari et al., 2021) (Figure 3D). Analogously, $d_{w,\text{Q}}^{\text{NL}}$ and $d_{a,\text{Q}}^{\text{NL}}$ were computed from QRS complexes (Bukhari et al., 2022a). Supplementary Figure S1 in the Supplementary Material shows linear and non-linear time warping for QRS complexes.

The set of all morphology-based T wave and QRS complex markers analyzed in this study included

• $d_{w,\text{T}}^{\text{u}}$ , representing temporal variations in T wave morphology (expressed in ms),
• *d*_a,T_, representing amplitude variations in T wave morphology (expressed as a %),
• $d_{w,\text{T}}^{\text{NL}}$ , representing nonlinear temporal variations in T wave morphology (expressed in ms),
• $d_{a,\text{T}}^{\text{NL}}$ , representing non-linear amplitude variations in T wave morphology (expressed as a %).
• $d_{\text{w},\text{Q}}^{\text{u}}$ , representing temporal variations in QRS morphology (expressed in ms),
• *d*_a,Q_, representing amplitude variations in QRS morphology (expressed as a %),
• $d_{w,\text{Q}}^{\text{NL}}$ , representing non-linear temporal variations in QRS morphology (expressed in ms), and
• $d_{a,\text{Q}}^{\text{NL}}$ , representing non-linear amplitude variations in QRS morphology (expressed as a %).

### 2.4 Effects of [K^+^], [Ca^2+^] and their combination on simulated T waves and QRS complexes

To assess the relationship between [K^+^], [Ca^2+^] and their combination with T wave and QRS complex characteristics in each model of the population, simulations were conducted under varying values of the electrolytes. The range of simulated [K^+^] values included the default level in the Ten Tusscher-Panfilov model, i.e., [K^+^] = 5.4 mM, as well as other levels below and above it: [K^+^] ∈ {3, 4, 5.4, 6.2} mM. In the case of [Ca^2+^], the range of simulated values included the default level of 2 mM and values around it: [Ca^2+^] ∈ {1.4, 2, 2.6, 3.2} mM. The combinations of [K^+^] and [Ca^2+^] included: (3, 3.2), (4, 2.6), (5.4, 2.0), and (6.2, 1.4) mM. The simulated ranges are similar to those observed in patients during HD (Palmieri et al., 2021a; Bukhari et al., 2021; Bukhari et al., 2022b).

Linear Pearson correlation analysis was also performed to assess the effects of [K^+^] and [Ca^2+^] on each investigated T wave and QRS complex marker.

### 2.5 Sensitivity analysis for assessment of inter-individual variability sources

Sensitivity analysis was performed to quantify how the proportion of endocardial, midmyocardial and epicardial cell layers modulated inter-individual variability in simulated T wave and QRS complex morphology markers at different [K^+^], [Ca^2+^] or their combination levels.

For each T wave and QRS complex marker at each given concentration of [K^+^] ([Ca^2+^] or both [K^+^] and [Ca^2+^], respectively), the percentage of change $D_{Y;c;a_{i}}$ in marker *Y* and its sensitivity *S*_*Y*;*c*;*a*1,*a*2_ to changes in the proportion of cells was computed as follows (Romero et al., 2009; Bukhari et al., 2019; Bukhari et al., 2021):

$$D_{Y;c;a_{i}}=\left(\frac{Y_{c;a_{i}}-Y_{\text{control}}}{Y_{\text{control}}}\right)\cdot 100,\;i\in \left\{1,2\right\}$$ (9)

$$S_{Y;c;a_{1},a_{2}}\;=\frac{\left(D_{Y;c;a_{2}}\;-\;D_{Y;c;a_{1}}\right)100}{a_{2}-a_{1}},$$ (10)

where *Y*_c;a_ is computed by averaging the changes for all possibles combinations having a percentage *a*_*i*_ of cell layer *c*, *c* ∈ {endo, mid, epi}, with respect to the reference taken from case *C*532 (*Y*_control_). The values of *a*_1_ and *a*_2_ were taken as the minimum and maximum proportions of cells in each layer, respectively: 10% and 50% for endocardial and midmyocardial cells, and 20% and 60% for epicardial cells.

## 3 Results

### 3.1 Evaluation of T wave and QRS complex changes induced by [K^+^] and [Ca^2+^] variations in heart-torso simulations

**FIGURE 4:**
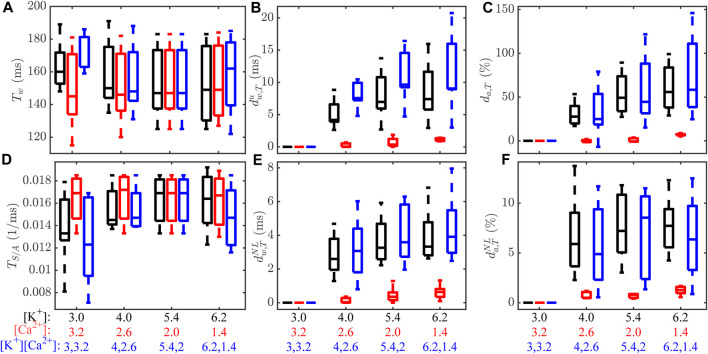
**(A–F)** Changes in *T*_w_, *T*_S/A_, $d_{w,\text{T}}^{\text{u}}$ , *d*_a,T_, $d_{w,\text{T}}^{\text{NL}}$ and $d_{a,\text{T}}^{\text{NL}}$ for varying [K^+^] at fixed [Ca^2+^] = 2.0 mM (black), varying [Ca^2+^] at fixed [K^+^] = 5.4 mM (red) and the combination of [K^+^] and [Ca^2+^] (blue), for ECGs simulated from the population of models. Central lines indicate the median, whereas bottom and top edges show the 25th and 75th percentiles, respectively.

T wave markers (*T*_w_, *T*_S/A_, $d_{w,\text{T}}^{\text{u}}$ , *d*_a,T_, $d_{w,\text{T}}^{\text{NL}}$ , $d_{a,\text{T}}^{\text{NL}}$ ) computed from simulated ECGs at varying [K^+^], [Ca^2+^] and their combinations are shown in Figure 4. The morphological markers $d_{w,\text{T}}^{\text{u}}$ , *d*_a,T_, $d_{w,\text{T}}^{\text{NL}}$ and $d_{a,\text{T}}^{\text{NL}}$ changed markedly at varying [K^+^] and [Ca^2+^]. Large differences between models in the population could be observed for all analyzed T wave markers. Supplementary Figure S2 in the Supplementary Material shows changes in QRS complex markers at varying [K^+^] and [Ca^2+^] and their combinations, with high variability between models for all markers.

### 3.2 Comparison of [K^+^]- and [Ca^2+^]-induced changes in T wave and QRS complex characteristics in simulations and patients

**FIGURE 5:**
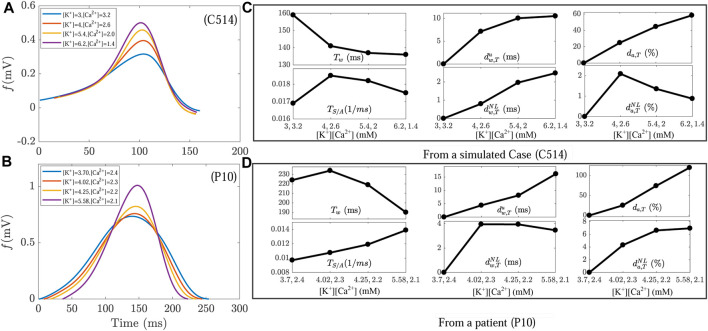
**(A,B)** T waves at varying [K^+^] and [Ca^2+^], for a simulated case (*C*514) and for a patient (P10). **(C,D)** Changes in T wave markers *T*_w_, *T*_S/A_, $d_{w,\text{T}}^{\text{u}}$ , *d*_a,T_, $d_{w,\text{T}}^{\text{NL}}$ and $d_{a,\text{T}}^{\text{NL}}$ for the same simulated case and patient, respectively. Note the different scales between the plots.

Figure 5 shows T waves and the analyzed markers *T*_w_, *T*_S/A_, $d_{w,\text{T}}^{\text{u}}$ , *d*_a,T_, $d_{w,\text{T}}^{\text{NL}}$ and $d_{a,\text{T}}^{\text{NL}}$ , computed from the ECGs of a particular model, *C*514, and a particular patient, P10, when concomitantly varying [K^+^] and [Ca^2+^]. More peaked T waves can be observed with increasing [K^+^] and decreasing [Ca^2+^] in both the model and the patient. Analogously, Supplementary Figure S3 in the Supplementary Material shows QRS complexes and analyzed QRS markers for a simulated case and a patient for simultaneous variations in [K^+^] and [Ca^2+^].

**FIGURE 6:**
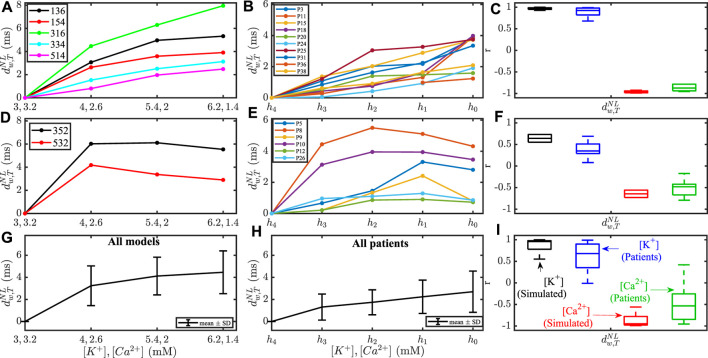
**(A,B,D,E,G,H)** Changes in $d_{w,\text{T}}^{\text{NL}}$ at varying [K^+^] and [Ca^2+^], in simulated cases and patients. **(C)** Pearson correlation coefficients, *r*, of $d_{w,\text{T}}^{\text{NL}}$ with [K^+^] and [Ca^2+^] for the simulated cases shown in **(A)** and the patients shown in **(B)**. **(F)** Pearson correlation coefficient *r* for the simulated cases shown in **(D)** and the patients shown in **(E)**. **(I)** correlation coefficient *r* for all the simulated cases and all the patients. *h*_0_–*h*_4_ are the HD time points corresponding to the onset and end of HD (*h*_4_ with lowest [K^+^] and highest [Ca^2+^] and *h*_0_ with highest [K^+^] and lowest [Ca^2+^].

Figure 6 shows a comparison of the changes in the marker $d_{w,\text{T}}^{\text{NL}}$ when varying both [K^+^] and [Ca^2+^] in the simulated cases and the patients. As can be observed by comparing panels a and b, panels d and e and panels g and h, the models in the population reproduced some specific patterns of change of $d_{w,\text{T}}^{\text{NL}}$ in the patients, albeit with some quantitative differences. These results were confirmed by computation of correlation coefficients, as shown in panels c, f and i of the same figure. The ability of our *in silico* population to reproduce $d_{w,\text{T}}^{\text{NL}}$ trends measured in some of the patients was equally valid for other T wave markers even if they did not present as remarkable changes as $d_{w,\text{T}}^{\text{NL}}$ when varying [K^+^] and [Ca^2+^]. Analogous results in simulated and patients’ ECGs are depicted in Supplementary Figure S4 of the Supplementary Material for the QRS-based $d_{a,\text{Q}}^{\text{NL}}$ , which was the one showing the largest changes in response to electrolyte variations.

**FIGURE 7:**
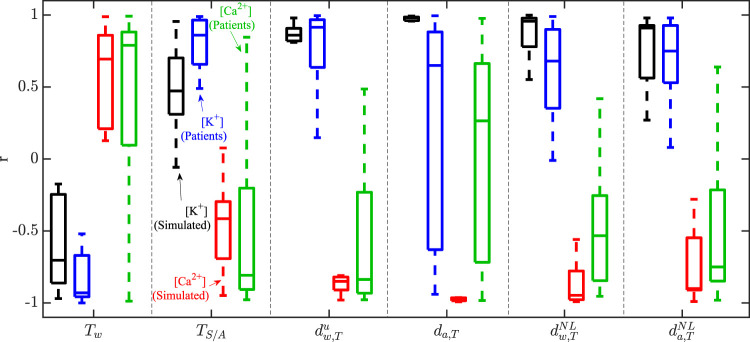
Pearson correlation coefficients, *r*, between each T wave marker (*T*_w_, *T*_S/A_, $d_{w,\text{T}}^{\text{u}}$ , *d*_a,T_, $d_{w,\text{T}}^{\text{NL}}$ and $d_{a,\text{T}}^{\text{NL}}$ ) and [K^+^] (black for simulated cases and blue for patients) or [Ca^2+^] (red for simulated cases and green for patients), for simultaneous variations in [K^+^] and [Ca^2+^].

To assess the extent to which our population of models could reproduce the inter-patient variability in T wave markers at concomitantly varying electrolyte levels, a correlation analysis was performed. Figure 7 shows the Pearson correlation coefficient *r* between each T wave marker and [K^+^], or [Ca^2+^], in the simulated and the patients’ ECGs. *T*_w_, $d_{w,\text{T}}^{\text{u}}$ and $d_{a,\text{T}}^{\text{NL}}$ were the markers most strongly correlated with [K^+^] (median *r* being −0.70, 0.87, 0.91 in simulations and −0.92, 0.93, 0.75 in patients, respectively) and [Ca^2+^] (median *r* being 0.70, −0.85, −0.91 in simulations and 0.79, −0.84, −0.75 in patients, respectively). Inter-individual variability in the correlation coefficients associated with *T*_w_ and *T*_S/A_ was high in both simulations and patients. For all other T wave morphology markers, the variability between models only partly reproduced the variability between patients. Table 1 provides the results for the quantitative comparison between simulated and patients’ T wave markers, in terms of median and interquartile range of *r* with [K^+^] and [Ca^2+^]. As can be seen from the table, all the analyzed morphology-based T wave markers correlated strongly with [K^+^] and [Ca^2+^] in simulations and patients, with part of the inter-patient variability being reproduced by the models.

**TABLE 1:** Median (interquartile range) of Pearson correlation coefficient between T wave markers and each of [K^+^] and [Ca^2+^] in the simulated cases and in the patients at varying [K^+^], [Ca^2+^] and their combination.

	*T*_w_	*T*_S/A_	$d_{w,\text{T}}^{\text{u}}$	*d*_a,T_	$d_{w,\text{T}}^{\text{NL}}$	$d_{a,\text{T}}^{\text{NL}}$
[K^+^] (Simul. [K^+^] only)	−0.86(0.56)	+0.64 (0.91)	+0.92 (0.04)	+0.97 (0.02)	+0.92 (0.11)	+0.89 (0.05)
[K^+^] (Simul. [K^+^] and [Ca^2+^])	−0.70 (0.52)	+0.47 (0.29)	+0.86 (0.07)	+0.98 (0.02)	+0.96 (0.14)	+0.91 (0.26)
[K^+^] (Patients)	−0.92 (0.28)	+0.84 (0.30)	+0.93 (0.31)	+0.65 (1.50)	+0.68 (0.53)	+0.75 (0.38)
[Ca^2+^] (Simul. [Ca^2+^] only)	−0.98 (0.16)	+0.56 (0.71)	−0.89 (0.06)	−0.72 (0.22)	−0.94 (0.02)	−0.89 (0.14)
[Ca^2+^] (Simul. [K^+^] and [Ca^2+^])	+0.70 (0.55)	−0.42 (0.29)	−0.86 (0.07)	−0.98 (0.02)	−0.95 (0.15)	−0.91 (0.25)
[Ca^2+^] (Patients)	+0.79 (0.76)	−0.81 (0.68)	−0.84 (0.68)	+0.27 (1.28)	−0.53 (0.59)	−0.75 (0.58)

Supplementary Figure S5 and Supplementary Table S2 in the Supplementary Material show correlation coefficients between QRS complex markers and electrolyte levels in simulated and patients’ ECGs.

### 3.3 Contribution of ventricular wall composition to inter-individual variability in T wave and QRS complex response to [K^+^] and [Ca^2+^] variations

The results of the sensitivity analysis performed to investigate how different proportions of endocardial, midmyocardial and epicardial cells may explain individual T wave responses when varying both [K^+^] and [Ca^2+^] are presented in Table 2 for all the analyzed simulated T wave markers. The highest sensitivity values were observed for morphology-based T wave markers, particularly $d_{w,\text{T}}^{\text{u}}$ and $d_{w,\text{T}}^{\text{NL}}$ , when varying the proportion of epicardial cells. Supplementary Table S2 in the Supplementary Material shows sensitivity results for QRS complex markers.

**TABLE 2:** Results of the sensitivity analysis, $S_{Y;c;a_{1},a_{2}}$ , for different values of combined [K^+^] and [Ca^2+^] for T wave markers, when varying cell proportions in layer *c* from *a*_1_ to *a*_2_ for human-specific Torso model.

$S_{Y;c;a_{1},a_{2}}$	*Y*	*T*_w_	*T*_S/A_	$d_{w,\text{T}}^{\text{u}}$	*d*_a,T_	$d_{w,\text{T}}^{\text{NL}}$	$d_{a,\text{T}}^{\text{NL}}$
*c*, *a*_1_, *a*_2_	[K^+^], [Ca^2+^]	%	%	%	%	%	%
Endo, 10, 50	4, 2.6	−0.36	1.26	−17.77	14.87	−2.16	5.32
	6.2, 1.4	0.98	−2.52	−67.62	12.45	−16.62	1.17
Mid, 10, 50	4, 2.6	6.40	−3.24	−14.32	1.06	10.13	−4.19
	6.2, 1.4	4.56	−1.16	−43.50	2.22	−4.26	7.42
Epi, 20, 60	4, 2.6	−6.03	1.98	32.09	−15.93	−7.98	−1.13
	6.2, 1.4	−5.55	3.68	111.13	−14.67	20.88	−8.59

## 4 Discussion

In our whole-heart and torso simulations, T wave and QRS complex duration, amplitude and morphology changed with [K^+^] and [Ca^2+^] in the same direction and with similar averaged magnitude as in ECGs from ESRD patients undergoing HD. In both simulations and patients, high inter-individual ECG variability was observed, which was accentuated at high [K^+^] and low [Ca^2+^]. Differences in cell type distribution, particularly in the proportion of epicardial cells, may explain inter-patient variability in T wave and QRS complex response to electrolyte variations within this model.

### 4.1 *In silico* heart-torso models reproduce [K^+^]- and [Ca^2+^]-induced changes in T wave and QRS complex measured in ESRD patients

We measured commonly used QRS complex and T wave markers describing characteristics related to their duration and amplitude, including T wave and QRS complex widths (*T*_w_, *QRS*_w_), QRS complex amplitude (*QRS*_a_) and T wave slope-to-amplitude ratio (*T*_S/A_), as well as our recently proposed morphological variability-based markers ( $d_{w,\text{T}}^{\text{u}}$ , $d_{\text{w},\text{Q}}^{\text{u}}$ , *d*_a,T_, *d*_a,Q_, $d_{w,\text{T}}^{\text{NL}}$ , $d_{w,\text{Q}}^{\text{NL}}$ , $d_{a,\text{T}}^{\text{NL}}$ , $d_{a,\text{Q}}^{\text{NL}}$ ) (Ramírez et al., 2017; Palmieri et al., 2021a; Bukhari et al., 2021; Bukhari et al., 2022a). We evaluated these markers in simulated ECGs derived from coupled heart-torso models with different proportions of endocardial, midmyocardial and epicardial cells, at varying [K^+^] (3–6.2 mM), [Ca^2+^] (1.4–3.2 mM) and their combinations.

We simulated a range of [Ca^2+^] values to assess the impact on the ECG markers. In the patients, total serum calcium concentration ([Ca^2+^]) was measured, which has higher values than the corresponding ionized calcium concentration. The values of [Ca^2+^] in our analyzed ESRD patients were in the ranges reported in other studies (Siyam and Klachko, 2013; Janmaat et al., 2018; Noordam et al., 2019; Akter et al., 2022). Supplementary Table S1 in the Supplementary Material shows the [K^+^], [Ca^2+^] and RR interval values in the 29 patients. Changes in the RR interval (inverse of heart rate) from the start to the end of the HD session were not statistically significant [see Table 2 in Bukhari et al. (2021) for the effects of RR intervals on all the analyzed T wave markers and Table 2 in Bukhari et al. (2022a) for the effects of RR intervals on all the analyzed QRS markers in the patients]. Consequently, we decided not to explore the effects of other stimulation frequencies in our heart-torso simulations.

We found that most of our simulated QRS and T wave markers (all except for *QRS*_w_, *d*_a,T_ and *d*_a,Q_) presented a diversity of patterns in their relationships with [K^+^] and [Ca^2+^] that were in line with our observations in ESRD patients during and after HD (Palmieri et al., 2021a; Bukhari et al., 2021; Bukhari et al., 2022b; Bukhari et al., 2022b). Overall, the T wave morphology markers $d_{w,\text{T}}^{\text{u}}$ , $d_{w,\text{T}}^{\text{NL}}$ and $d_{a,\text{T}}^{\text{NL}}$ were the ones that most notably changed with [K^+^], somewhat less notably with [Ca^2+^] and very remarkably with their combination, in average over the population of *in silico* models (Figure 4). When we tested lower [Ca^2+^] values of 0.8, 1, and 1.2 mM, which would correspond to ranges of ionized calcium concentrations, the changes in the T wave markers were accentuated with respect to the ones observed for [Ca^2+^] varying from 1.4 to 3.2 mM, as illustrated in Supplementary Figure S6 of the Supplementary Material. Nevertheless, the variations in the T waves when only varying [Ca^2+^] were still smaller than the ones measured when varying [K^+^] and [Ca^2+^] simultaneously. Our simulation results were in line with averaged changes presented by the analyzed markers in ESRD patients, as shown here and in previous studies where the markers were first evaluated during HD (Palmieri et al., 2021a; Bukhari et al., 2021; Bukhari et al., 2022b). This generally corresponded to more peaked T waves, with lower amplitude and/or longer duration, which presented increasingly larger morphological differences with respect to a reference T wave (calculated at physiological electrolyte levels) when [K^+^] was increased and [Ca^2+^] was decreased (Figure 5) (Palmieri et al., 2021a; Bukhari et al., 2021; Bukhari et al., 2022b). Regarding the QRS complex, markers describing amplitude characteristics, like *QRS*_a_ and $d_{a,\text{Q}}^{\text{NL}}$ , also varied appreciably with [K^+^], [Ca^2+^] and, particularly, with their combination, in both the simulations and the patients. Typically, the averaged QRS complex in the simulated and patient populations became lower in amplitude and had larger non-linear amplitude morphological variations than a reference physiological QRS complex when evaluated in response to increasing [K^+^] and decreasing [Ca^2+^] (Supplementary Figure S3 in the Supplementary Material) (Bukhari et al., 2022a).

For confirmation of the ability of our *in silico* population to describe averaged trends of T wave and QRS complex changes with electrolyte variations in patients, a correlation analysis was performed. Among the analyzed T wave markers, $d_{w,\text{T}}^{\text{u}}$ and $d_{a,\text{T}}^{\text{NL}}$ were the most strongly correlated with [K^+^] and [Ca^2+^] in both the simulations and the patients (absolute median correlation coefficient ranging from 0.75 to 0.93). In the case of QRS complex markers, *QRS*_a_, $d_{w,\text{Q}}^{\text{NL}}$ and $d_{a,\text{Q}}^{\text{NL}}$ were the ones most strongly associated with [K^+^] and [Ca^2+^] in the simulations and the patients (absolute median correlation coefficient ranging from 0.71 to 0.98). These results support the use of our *in silico* population of models to describe the average response of T wave and QRS complex to variations in electrolyte levels like those seen in ESRD patients during and after HD.

### 4.2 Differences in ventricular wall composition may explain inter-individual variability in [K^+^]- and [Ca^2+^]-induced changes in T wave and QRS complex

After assessing how our population of *in silico* models could reproduce the general trends of electrolyte-induced changes in ECG markers observed in ESRD patients, we investigated how it may explain the high inter-patient variability in such changes. In agreement with the diversity of patterns of QRS complex and T wave changes in response to [K^+^] and [Ca^2+^] variations measured in the patients, we found different ventricular models presenting largely distinct behaviors that covered some of the patterns described in the patients. Models with thin epicardial layers were mainly related with a non-monotonic behavior of the evaluated ECG markers similar to that presented by some ESRD patients. This was particularly clear for the T wave morphology marker $d_{w,\text{T}}^{\text{NL}}$ , illustrated in Figure 6. Most models with thick epicardial layers reproduced the increasing linear trend of $d_{w,\text{T}}^{\text{NL}}$ observed in many patients (Figure 6). For the QRS complex, we found that models with thick midmyocardial layers could replicate the increasing linear trend in the marker $d_{a,\text{Q}}^{\text{NL}}$ measured in some patients (Supplementary Figure S4 of Supplementary Material). Also, the non-monotonic behavior of $d_{a,\text{Q}}^{\text{NL}}$ in many other patients was obtained for models with thick epicardial layers (Supplementary Figure S4 of the Supplementary Material). Therefore, we may conclude that the different transmural heterogeneities simulated in this study may explain the inter-patient variability in the ECG response to [K^+^] and [Ca^2+^] variations.

To quantify how different ventricular wall compositions may explain individual ECG responses at a range of [K^+^] and [Ca^2+^], we computed QRS and T wave marker sensitivities to variations in the thickness of the endocardial, midmyocardial and epicardial cell layers. The highest sensitivity values were observed for T wave morphology markers $d_{w,\text{T}}^{\text{u}}$ , $d_{w,\text{T}}^{\text{NL}}$ and $d_{a,\text{T}}^{\text{NL}}$ and for QRS morphology markers $d_{w,\text{Q}}^{\text{NL}}$ , *d*_a,Q_ and $d_{a,\text{Q}}^{\text{NL}}$ . Extremely high sensitivities were found for some of these T wave markers at abnormally high levels of [K^+^] and low levels of [Ca^2+^], which may explain the remarkably large inter-patient variability observed at the beginning of the HD, when [K^+^] was enhanced and [Ca^2+^] was reduced. In particular, $d_{w,\text{T}}^{\text{u}}$ very notably increased for progressively larger proportions of epicardial cells and it decreased for progressively larger proportions of endocardial or midmyocardial cells. The largest sensitivity of $d_{w,\text{T}}^{\text{u}}$ to variations in the proportion of epicardial cells within the ventricular wall agrees with previous reports showing the contribution of epicardial cells to other forms of repolarization variability like T wave alternans (Janusek et al., 2018). For [K^+^] = 6.2 mM and [Ca^2+^] = 1.4 mM, sensitivity values above 110% were found, which corresponded to a coefficient of determination of 0.93 for the relationship between $d_{w,\text{T}}^{\text{u}}$ and the proportion of epicardial cells. Previous experimental and theoretical studies have described how cell type distributions influence ECG characteristics (Hooft van Huysduynen et al., 2005; Okada et al., 2011; Rivolta et al., 2015; Ramírez et al., 2017; Janusek et al., 2018; Srinivasan et al., 2019). In particular, Janusek et al. (2018) reported that the contribution of epicardial cells to T wave alternans was significantly higher than that of midmyocardial cells, which would be in line with our results for T wave morphological variability. In Rivolta et al. (2015), cell type distributions were shown to highly affect both repolarization and T wave morphology parameters, in concordance with our findings on the impact of ventricular wall composition on T wave characteristics, particularly at abnormal [K^+^] and [Ca^2+^] values.

For the above described analysis, we included different proportions of endocardial, midmyocardial and epicardial layers that comprised those used in previous simulation studies, which were in turn based on experimentally reported transmural cell proportions (Drouin et al., 1995; Gima and Rudy, 2002; Ten Tusscher and Panfilov, 2006; Bueno-Orovio et al., 2008; Pueyo et al., 2010; Pueyo et al., 2011). In particular, the following proportions were used for endocardial, midmyocardial and epicardial layers in previous simulation studies: Gima and Rudy (2002) used 4:2:4; Bueno-Orovio et al. (2008) used 5:3:2 and Pueyo et al. (2010) used 3:3:4. In a previous study (Bukhari et al., 2021), we included larger ranges of cell type proportions in simulated 1D fibers, with the endocardial percentage ranging from 10% to 50%, the midmyocardial percentage from 10% to 50% and the epicardial percentage from 20% to 80%. Here, we included a subset of those cases, which covered the proportions most commonly employed in previous simulation studies. Even if we showed that variability in the thickness of endocardial, midmyocardial and epicardial layers may be a cause for the observed inter-individual variability in the ECG response to electrolyte variations, this was based on the large range of cell type proportions simulated in our study. If the variation in the myocardial cell distribution in the patients were substantially smaller, the differences in ventricular wall distribution might play a less relevant role in the observed inter-patient variability of the ECG response to electrolyte variations. Moreover, it is not even known whether M cells with distinct electrophysiological properties exist or not. There has been and there is still controversy regarding these cells, with some studies showing evidence of their existence (Liu et al., 1993; Drouin et al., 1995; Anyukhovsky et al., 1996; Baláti et al., 1998; Stankovicova et al., 2000; Yan et al., 2003; Wilson et al., 2011) and others questioning it, particularly in intact hearts (Rodríguez-Sinovas et al., 1997; Bryant et al., 1998; Conrath et al., 2004; Janse et al., 2012).

Our *in silico* analysis could pave the path to understand how patients with different ventricular wall compositions may present largely different responses to serum electrolyte variations, even if other factors, such as interventricular, apicobasal or anteroposterior differences (Janse et al., 2012; Opthof et al., 2017), might also explain inter-patient variability. These results could help to improve monitoring serum ion concentrations and predicting arrhythmic events in ESRD patients based on investigations combining *in silico* modeling and simulation with signal processing of the ECG.

### 4.3 Related work

Several previous studies have characterized ECG features in relation to electrolyte concentrations. Hernández Mesa et al. (2018) and Pilia et al. (2017) computed ECGs at different [Ca^2+^] from the ventricular electrophysiology and a torso model. No changes with [Ca^2+^] were found in QRS duration, while the R wave amplitude and energy diminished with decreasing [Ca^2+^] (Hernández Mesa et al., 2018), which would agree with our observations of reduced *QRS*_a_ and $d_{a,\text{Q}}^{\text{NL}}$ . Also, the T wave slopes were reported to increase with decreasing [Ca^2+^] (Hernández Mesa et al., 2018) and increasing [K^+^] (Pilia et al., 2017), which is in line with our observations of more peaked T waves at low [Ca^2+^] and high [K^+^].

Other studies have simulated human ventricular electrophysiology in a one-dimensional transmural model and have derived pseudo-ECGs at varying [K^+^] (Kharche et al., 2012; Corsi et al., 2017). Increases in T wave slope were reported in association with hyperkalemia, which is in agreement with our results.

Inter-patient variability in the relation between electrolytes and ECG markers has been reported by several authors (Buemi et al., 2005; Montague et al., 2008; Severi et al., 2009b; Alabd et al., 2011; Green et al., 2013; Corsi et al., 2017; Yoon et al., 2018; Pilia et al., 2020; Palmieri et al., 2021b; Palmieri et al., 2022). We are not aware of previous work explaining this variability in terms of variations in ventricular wall composition, except for our previous study in simulated ventricular 1D fibers (Bukhari et al., 2021). By extending our investigations from 1D fibers to 3D ventricular and torso models, we could compute ECG features in a more realistic way that allowed for a more accurate comparison with patient data.

### 4.4 Study limitations and future research

Our simulations were performed with the most realistic available techniques to model the electrophysiology of the heart and the resulting ECGs. The electrophysiology was represented by a widely-used and detailed model of the human ventricular myocyte (Ten Tusscher and Panfilov, 2006) that is thoroughly rooted in experimental data. We included the updates to the Ten Tusscher-Panfilov model proposed in Severi et al. (2009a) so that the model reproduced a physiological APD-[Ca^2+^] relationship as described in several *in vitro* and *in vivo* studies (Temte and Davis, 1967; Leitch and Brown, 1996; Bai et al., 2005). This model allowed running cell and tissue simulations at an affordable computational time. Initially, we considered the O’Hara-Rudy model (O’Hara et al., 2011) and the Grandi-Pasqualini-Bers model (Grandi et al., 2010), but these models had limitations with respect to the simulation of hyperkalemic effects in tissues, as they did not show propagation of excitation for [K^+^] values larger than 6 and 8 mM, respectively (Dutta et al., 2017). Subsequently, we considered the modified version of the O’Hara-Rudy model proposed by Dutta et al. (2017), but we observed that this model was not able to reproduce the inverse relationship between APD and [Ca^2+^] reported experimentally. Another model recently proposed by Bartolucci et al. (2020) which is a modified version of the O’Hara-Rudy 2011 model, can reproduce a physiological APD-[Ca^2+^] relationship but requires further testing in 3D tissues. The Bartolucci et al. model could be a good option for future studies, considering its ability to reproduce a physiological APD-[Ca^2+^] relationship.

We found that variations in wall composition may explain inter-patient variability in the ECG response to electrolyte changes. While we have shown that variability in the thickness of endocardial, midmyocardial and epicardial layers may be a cause, we cannot exclude additional contributing factors. On top of including other types of ventricular heterogeneities, as discussed above, future studies could also investigate electrophysiological differences across the ventricular wall, e.g., by incorporating transmural differences in the *I*_CaL_ formulation. In any case, the findings of our study remain to be confirmed. If confirmed, this could imply that accurate serum ion concentration estimation in individual patients would require *a priori* knowledge of the individual patient’s cell type distribution. Methods could be developed to infer such distribution from the ECG and blood data acquired from the patient during one or several prior HD sessions. ECG-based estimators of serum ion concentrations could be subsequently derived as a function of the patient’s cell type distribution.

Our *in silico* population included 7 whole-ventricle models with different cell type distributions across the ventricular wall, which we used to simulate variations in [K^+^] from 3 to 6.2 mM and in [Ca^2+^] from 1.4 to 3.2 mM. Future studies could extend the methods proposed here to add larger numbers of coupled ventricle-torso models and simulate a wider range of [K^+^] and [Ca^2+^].

The effects of other electrolyte concentrations such as magnesium, [Mg^2+^], which has been reported to be possibly involved in observed alterations in the ECG (Noordam et al., 2019; Naksuk et al., 2017; Jhang et al., 2013; van den Bergh et al., 2004), could be accounted for in the simulations if information on the variation of these electrolytes during and after HD were available. In the present study, we did not investigate [Mg^2+^] variations because serum [Mg^2+^] levels were not measured in our patients.

To investigate other sources of inter-individual variability in the ECG response to serum electrolyte variations, future studies could include other ventricular heterogeneities on top of transmural ones, like interventricular, apicobasal or anteroposterior (Janse et al., 2012; Opthof et al., 2017), which might play a relevant role in determining ECG characteristics under normal and abnormal electrolyte concentrations.

## 5 Conclusion

Our *in silico* population of coupled ventricle-torso models with different ventricular wall compositions allows to determine patient-dependent responses of T wave and QRS complex to variations in [K^+^] and [Ca^2+^]. Differences in the proportion of ventricular cell types, particularly of epicardial cells, may explain the inter-subject variability in such responses. These findings can pave a path to design better tools for non-invasive serum electrolyte monitoring and prediction of arrhythmic events in the patients.

## Data Availability

The original contributions presented in the study are included in the article/Supplementary Material, further inquiries can be directed to the corresponding author.
