# Supplementary material for: Differences in ventricular wall composition may explain inter-patient variability in the ECG response to variations in serum potassium and calcium
Source: Front Physiol. 2023 Oct 11;14:1060919. doi: 10.3389/fphys.2023.1060919 (PMC10598848; doi:10.3389/fphys.2023.1060919)
Supplement: Supplementary file 1 [file DataSheet1.pdf]

# Supplementary Material

## 1 COMPUTATION OF QRS MORPHOLOGY MARKERS

Linear and non-linear time warping of QRS complexes for a particular simulated case ($C334$) are shown in Figure S1 at particular $[\mathrm{K}^+]$ and $[\mathrm{Ca}^{2+}]$.

## 2 EVALUATION OF QRS COMPLEX CHANGES INDUCED BY $[\mathrm{K}^+]$ AND $[\mathrm{Ca}^{2+}]$ VARIATIONS IN SIMULATIONS

QRS complex markers ($QRS_{\mathrm{w}}$, $QRS_{\mathrm{a}}$, $d^{\mathrm{u}}_{\mathrm{w,Q}}$, $d_{\mathrm{a,Q}}$, $d^{\mathrm{NL}}_{\mathrm{w,Q}}$, $d^{\mathrm{NL}}_{\mathrm{a,Q}}$) computed from simulated ECGs at varying $[\mathrm{K}^+]$, $[\mathrm{Ca}^{2+}]$ and their combinations are shown in Figure S2. All the analyzed markers vary significantly at varying $[\mathrm{K}^+]$ and $[\mathrm{Ca}^{2+}]$.

## 3 COMPARISON OF INTER-INDIVIDUAL VARIABILITY IN QRS COMPLEX CHARACTERISTICS AT VARYING $[\mathrm{K}^+]$ AND $[\mathrm{Ca}^{2+}]$ IN SIMULATIONS AND PATIENTS

Figure S3 shows QRS complexes and the analyzed markers $QRS_{\mathrm{w}}$, $QRS_{\mathrm{a}}$, $d^{\mathrm{u}}_{\mathrm{w,Q}}$, $d_{\mathrm{a,Q}}$, $d^{\mathrm{NL}}_{\mathrm{w,Q}}$ and $d^{\mathrm{NL}}_{\mathrm{a,Q}}$, computed from the ECGs of a particular model, $C514$, and a particular patient, P10, when concomitantly varying $[\mathrm{K}^+]$ and $[\mathrm{Ca}^{2+}]$. More peaked positive QRS complexes can be observed with decreasing $[\mathrm{K}^+]$ and increasing $[\mathrm{Ca}^{2+}]$ in both the model and the patient.

Figure S4 shows a comparison of the changes in the marker $d^{\mathrm{NL}}_{\mathrm{a,Q}}$ when varying both $[\mathrm{K}^+]$ and $[\mathrm{Ca}^{2+}]$ in the simulated cases and the patients. As can be observed by comparing panels a and b, panels d and e and panels g and h, the models in the population reproduced some specific patterns of change of $d^{\mathrm{NL}}_{\mathrm{a,Q}}$ in the patients, albeit with some quantitative differences. These results were confirmed by computation of Pearson correlation coefficients, as shown in panels c, f and i of the same figure. The ability of our *in silico* population to reproduce $d^{\mathrm{NL}}_{\mathrm{a,Q}}$ trends measured in some of the patients was equally valid for other QRS complex markers even if they did not present as remarkable changes as $d^{\mathrm{NL}}_{\mathrm{a,Q}}$ when varying $[\mathrm{K}^+]$ and $[\mathrm{Ca}^{2+}]$.

To assess the extent to which our population of models could reproduce the inter-patient variability in QRS complex markers at concomitantly varying electrolyte levels, a correlation analysis was performed. Figure S5 shows the Pearson correlation coefficient $r$ between each QRS complex marker and $[\mathrm{K}^+]$, or

**Table S1.** Median (interquartile range) with 75% (and 25%) percentile of $[\mathrm{K}^+]$, $[\mathrm{Ca}^{2+}]$ and RR variations in 29 ESRD patients.

| | $h_0$ | $h_1$ | $h_2$ | $h_3$ | $h_4$ |
|---|---|---|---|---|---|
| $[\mathrm{K}^+]$ (mM) [median(IQR)] | 5.10(1.30) | 3.90(0.86) | 3.63(0.81) | 3.40(0.71) | 3.40(0.56) |
| $[\mathrm{K}^+]$ (mM) [75% (25%) percentile] | 5.80(4.50) | 4.36(3.50) | 4.09(3.28) | 3.80(3.09) | 3.76(3.20) |
| $[\mathrm{Ca}^{2+}]$ (mM) [median(IQR)] | 2.15(0.20) | 2.23(0.20) | 2.29(0.19) | 2.31(0.23) | 2.36(0.21) |
| $[\mathrm{Ca}^{2+}]$ (mM) [75% (25%) percentile] | 2.23(2.03) | 2.32(2.13) | 2.36(2.17) | 2.40(2.17) | 2.40(2.19) |
| $RR$ (s) [median(IQR)] | 0.75(0.22) | 0.79(0.29) | 0.76(0.24) | 0.75(0.17) | 0.75(0.26) |
| $RR$ (s) [75% (25%) percentile] | 0.89(0.67) | 0.97(0.68) | 0.95(0.71) | 0.89(0.71) | 0.95(0.68) |

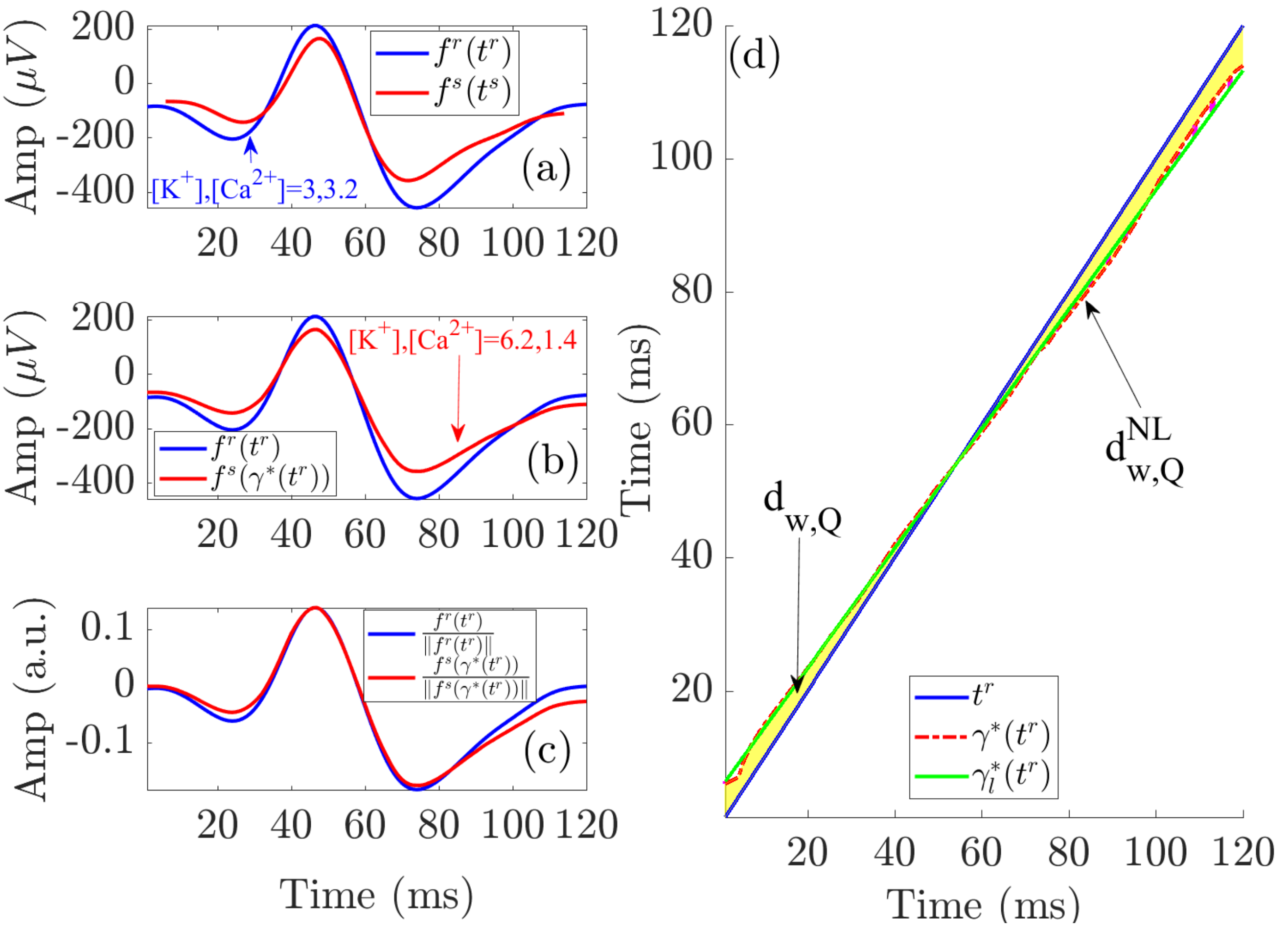


Figure S1: Linear and non-linear time warping for a particular simulated case ($C334$) from 3D torso-heart model. Panel (a) shows reference (blue) and investigated (red) QRS complexes obtained from a combination of $[\mathrm{K}^+]$ and $[\mathrm{Ca}^{2+}]$. Panel (b) shows the warped QRS complexes, which have the same duration while keeping the original amplitude. Panel (c) depicts the warped QRS complexes after normalization by their L2-norms. The area (yellow region) between both QRS complexes in panel (d) represents $d^{\mathrm{u}}_{\mathrm{w,Q}}$, which quantifies the total amount of warping. The green solid line is the linear regression function $\gamma^*_l(t^r)$ best fitted to $\gamma^*(t^r)$. The marker $d^{\mathrm{NL}}_{\mathrm{w,Q}}$ quantifies the non-linear warping by computing the area of the dashed magenta region between $\gamma^*(t^r)$ and $\gamma^*_l(t^r)$.

$[\mathrm{Ca}^{2+}]$, in the simulated and the patients' ECGs. $QRS_{\mathrm{a}}$, $d^{\mathrm{NL}}_{\mathrm{w,Q}}$ and $d^{\mathrm{NL}}_{\mathrm{a,Q}}$ were the markers most highly correlated with $[\mathrm{K}^+]$ (median $r$ being $-0.98$, $0.80$, $0.71$ in simulations, and $-0.86$, $0.86$, $0.87$ in patients, respectively) and $[\mathrm{Ca}^{2+}]$ (median $r$ being $0.98$, $-0.82$, $-0.71$ in simulations, and $0.82$, $-0.71$, $-0.80$ in patients, respectively). Inter-individual variability in the correlation coefficients associated with $QRS_{\mathrm{w}}$and $d_{\mathrm{a,Q}}$ was high in both simulations and patients. For all other QRS complex morphology markers, the variability between *in silico* models only partly reproduced the variability between patients.

Table S2 provides the results for the quantitative comparison between simulated and patients' QRS complex markers, in terms of median and interquartile range of Pearson correlation coefficient $r$ with $[\mathrm{K}^+]$ and $[\mathrm{Ca}^{2+}]$. As can be seen from the table, all the analyzed morphology-based QRS complex

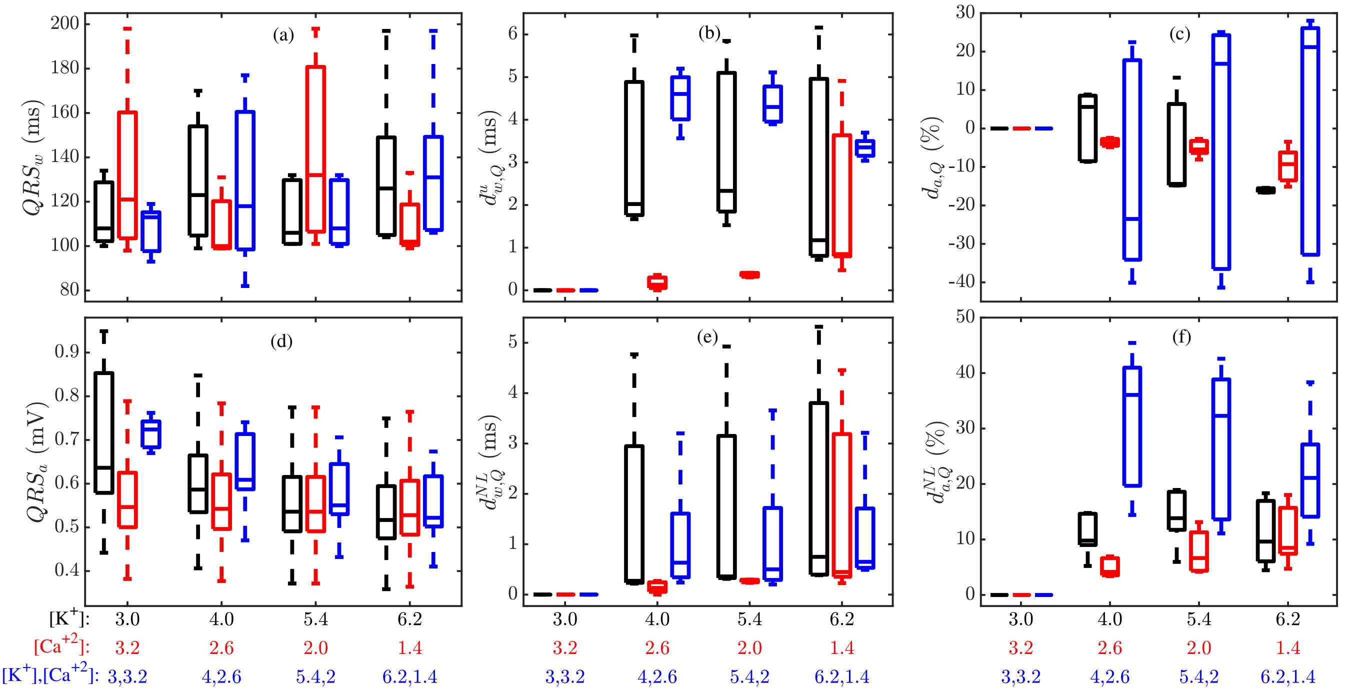


Figure S2: Panels a–f: Changes in $QRS_\mathrm{w}$, $QRS_\mathrm{a}$, $d^\mathrm{u}_\mathrm{w,Q}$, $d_\mathrm{a,Q}$, $d^\mathrm{NL}_\mathrm{w,Q}$ and $d^\mathrm{NL}_\mathrm{a,Q}$ for varying $[\mathrm{K}^+]$ at fixed $[\mathrm{Ca}^{2+}]$=2.0 mM (black), varying $[\mathrm{Ca}^{2+}]$ at fixed $[\mathrm{K}^+]$ =5.4mM (red), and the combination of $[\mathrm{K}^+]$ and $[\mathrm{Ca}^{2+}]$ (blue), for ECGs simulated from the population of models. Central lines indicate the median, whereas bottom and top edges show the 25th and 75th percentiles, respectively.

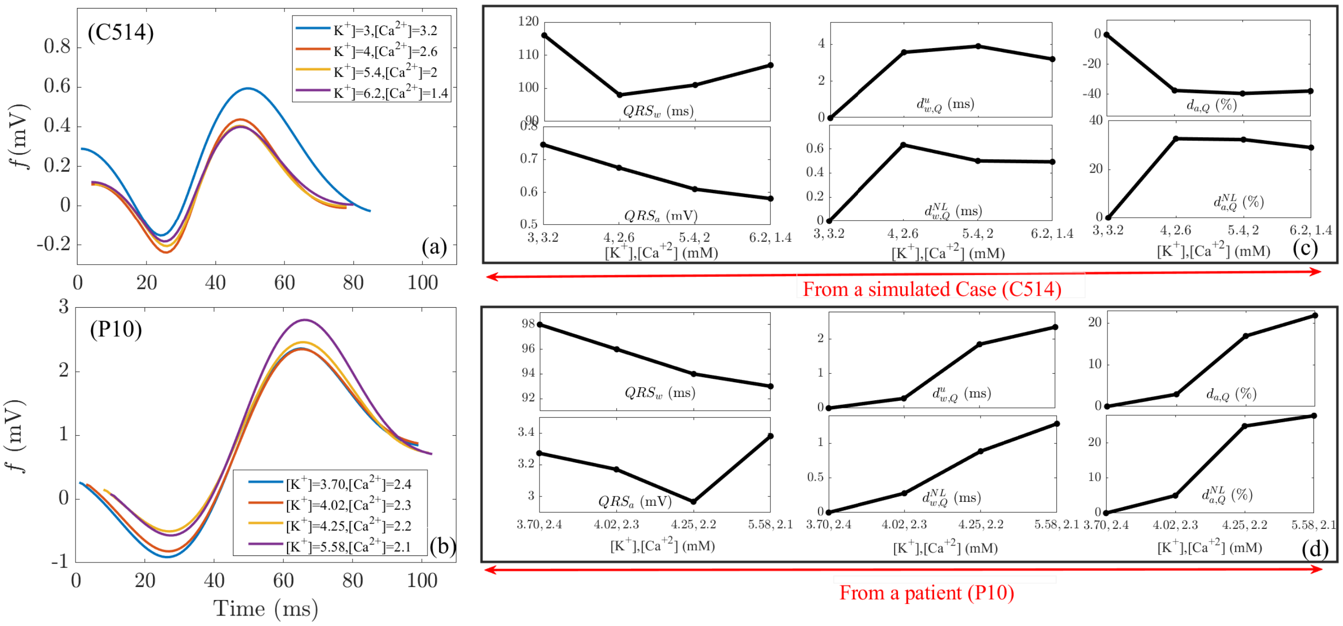


Figure S3: Panels a–b: QRS complexes at varying $[\mathrm{K}^+]$ and $[\mathrm{Ca}^{2+}]$, for a simulated case ($C514$) and for a patient (P10). Panels c–d: Changes in QRS complex markers $QRS_\mathrm{w}$, $QRS_\mathrm{a}$, $d^\mathrm{u}_\mathrm{w,Q}$, $d_\mathrm{a,Q}$, $d^\mathrm{NL}_\mathrm{w,Q}$ and $d^\mathrm{NL}_\mathrm{a,Q}$ for the same simulated case and patient, respectively. Note the different scales between the plots.

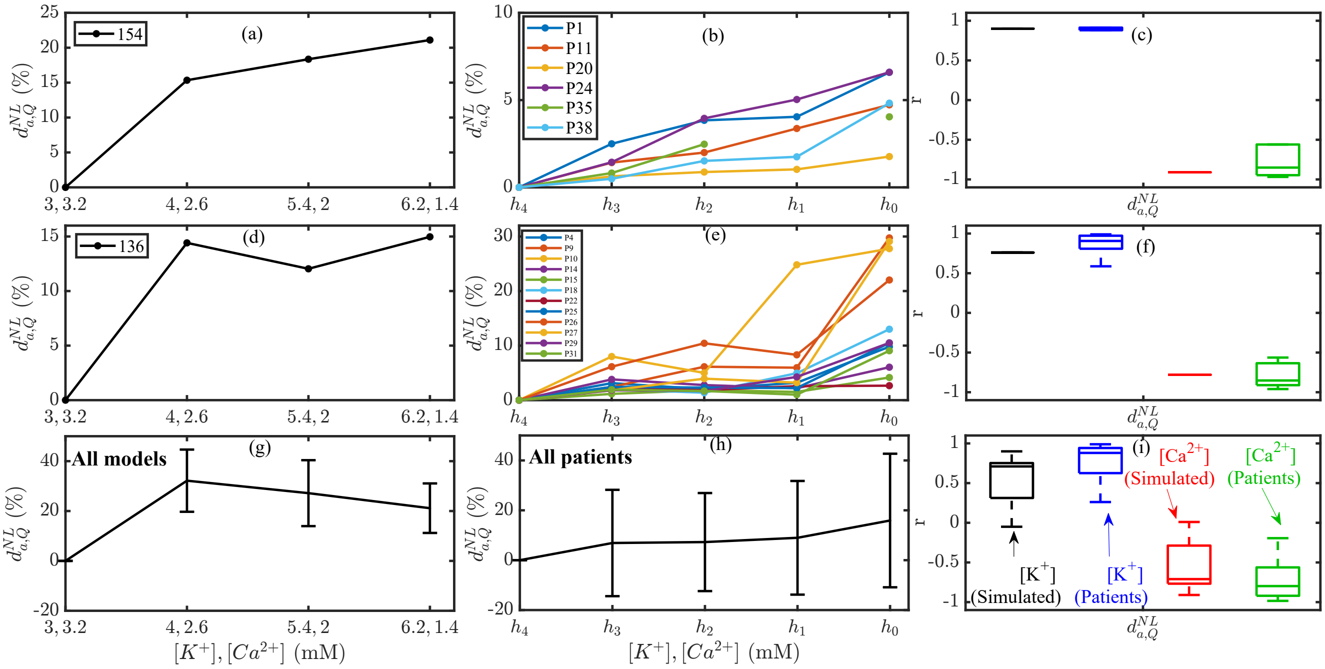


Figure S4: Panels a–b, d–e, g–h: Changes in $d^{\mathrm{NL}}_{\mathrm{a,Q}}$ at varying $[\mathrm{K}^+]$ and $[\mathrm{Ca}^{2+}]$, in simulated cases and patients. Panel c: Pearson correlation coefficients, $r$, of $d^{\mathrm{NL}}_{\mathrm{a,Q}}$ with $[\mathrm{K}^+]$ and $[\mathrm{Ca}^{2+}]$ for the simulated cases shown in a and the patients shown in b. Panel f: Pearson correlation coefficient $r$ for the simulated cases shown in d and the patients shown in e. Panel i: correlation coefficient $r$ for all the simulated cases and all the patients. $h_0$–$h_4$ are the HD time points corresponding to the onset and end of HD ($h_4$ with lowest $[\mathrm{K}^+]$ and highest $[\mathrm{Ca}^{2+}]$ and $h_0$ with highest $[\mathrm{K}^+]$ and lowest $[\mathrm{Ca}^{2+}]$.

**Table S2.** Median (interquartile range) of Pearson correlation coefficient between QRS complex markers and each of $[\mathrm{K}^+]$ and $[\mathrm{Ca}^{2+}]$ in the simulated cases and in the patients at varying $[\mathrm{K}^+]$, $[\mathrm{Ca}^{2+}]$ and their combination.

| | $QRS_{\mathrm{w}}$ | $QRS_{\mathrm{a}}$ | $d^{\mathrm{u}}_{\mathrm{w,Q}}$ | $d_{\mathrm{a,Q}}$ | $d^{\mathrm{NL}}_{\mathrm{w,Q}}$ | $d^{\mathrm{NL}}_{\mathrm{a,Q}}$ |
|---|---|---|---|---|---|---|
| $[\mathrm{K}^+]$ (Simul. $[\mathrm{K}^+]$ only) | −0.05(0.71) | −0.99(0.00) | 0.46(0.32) | −0.82(0.34) | 0.82(0.18) | 0.76(0.17) |
| $[\mathrm{K}^+]$ (Simul. $[\mathrm{K}^+]$ & $[\mathrm{Ca}^{2+}]$) | 0.66(0.86) | −0.98(0.03) | 0.58(0.09) | 0.35(1.33) | 0.80(0.10) | 0.71(0.37) |
| $[\mathrm{K}^+]$ (Patients) | −0.51(1.24) | −0.86(0.25) | 0.82(0.33) | −0.81(1.49) | 0.86(0.27) | 0.87(0.28) |
| $[\mathrm{Ca}^{2+}]$ (Simul. $[\mathrm{Ca}^{2+}]$ only) | −0.66(1.31) | 0.99(0.01) | −0.87(0.14) | 0.97(0.02) | −0.91(0.12) | −0.98(0.24) |
| $[\mathrm{Ca}^{2+}]$ (Simul. $[\mathrm{K}^+]$ & $[\mathrm{Ca}^{2+}]$) | −0.59(0.90) | 0.98(0.03) | −0.59(0.09) | −0.41(1.34) | −0.82(0.12) | −0.71(0.40) |
| $[\mathrm{Ca}^{2+}]$ (Patients) | 0.12(1.47) | 0.82(0.48) | −0.74(0.76) | 0.78(1.45) | −0.71(0.49) | −0.80(0.36) |

markers, except $QRS_{\mathrm{w}}$, correlated strongly with $[\mathrm{K}^+]$ and $[\mathrm{Ca}^{2+}]$ in simulations and patients, with part of the inter-patient variability being reproduced by *in silico* models.

## 4 CONTRIBUTION OF VENTRICULAR WALL COMPOSITION TO INTER-INDIVIDUAL QRS COMPLEX VARIABILITY AT VARYING $[\mathrm{K}^+]$ AND $[\mathrm{Ca}^{2+}]$

The results of the sensitivity analysis performed to investigate how different proportions of endocardial, midmyocardial and epicardial cells contribute to explain individual QRS complex responses when varying both $[\mathrm{K}^+]$ and $[\mathrm{Ca}^{2+}]$ are presented in Table S3 for all the analyzed simulated QRS markers. The highest

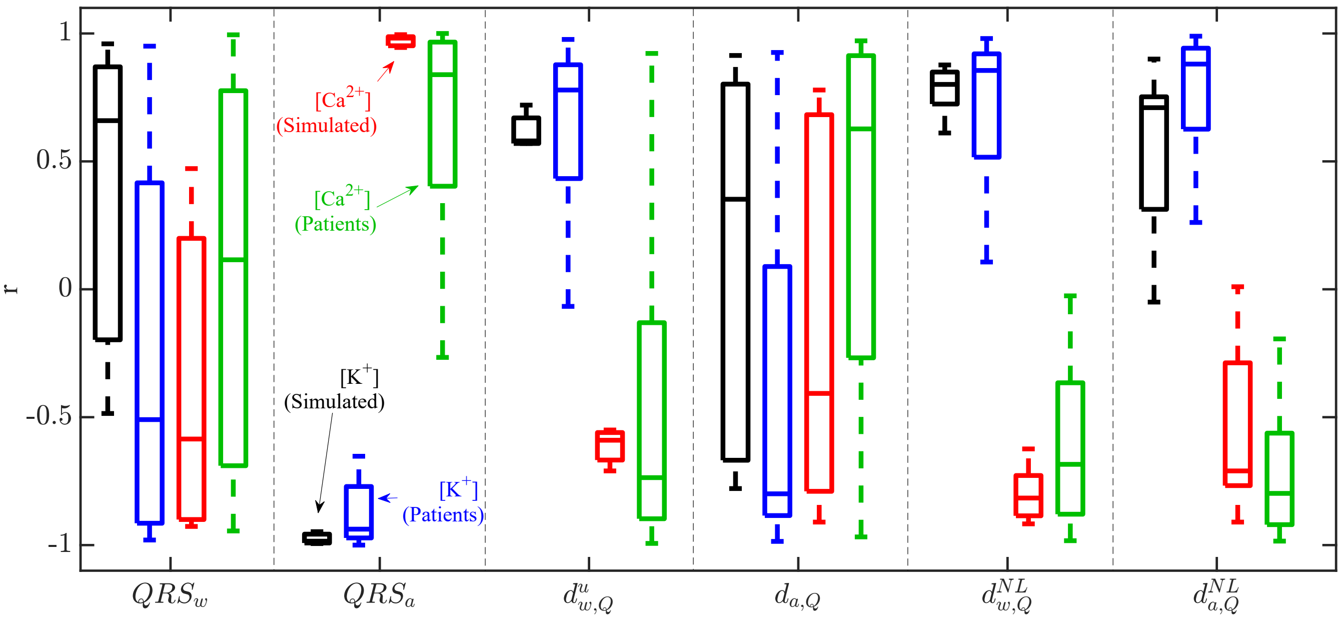


Figure S5: Pearson correlation coefficients, $r$, between each QRS complex marker ($QRS_\mathrm{w}$, $QRS_\mathrm{a}$, $d^\mathrm{u}_\mathrm{w,Q}$, $d_\mathrm{a,Q}$, $d^\mathrm{NL}_\mathrm{w,Q}$ and $d^\mathrm{NL}_\mathrm{a,Q}$) and $[\mathrm{K}^+]$ (black for simulated cases and blue for patients) or $[\mathrm{Ca}^{2+}]$ (red for simulated cases and green for patients), for simultaneous variations in $[\mathrm{K}^+]$ and $[\mathrm{Ca}^{2+}]$.

sensitivity values were observed for morphology-based QRS complex markers, particularly $d^\mathrm{NL}_\mathrm{w,Q}$ and $d_\mathrm{a,Q}$, when varying the proportion of endocardial or midmyocardial cells.

**Table S3.** Results of the sensitivity analysis, $S_{Y;c;a_1,a_2}$, for different values of combined $[\mathrm{K}^+]$ and $[\mathrm{Ca}^{2+}]$ for QRS complex markers, when varying cell proportions in layer $c$ from $a_1$ to $a_2$ for human-specific Torso model.

| $S_{Y;c;a_1,a_2}$ | $Y$ | $QRS_\mathrm{w}$ | $QRS_\mathrm{a}$ | $d^\mathrm{u}_\mathrm{w,Q}$ | $d_\mathrm{a,Q}$ | $d^\mathrm{NL}_\mathrm{w,Q}$ | $d^\mathrm{NL}_\mathrm{a,Q}$ |
|---|---|---|---|---|---|---|---|
| $c$, $a_1$, $a_2$ | $[\mathrm{K}^+]$,$[\mathrm{Ca}^{2+}]$ | % | % | % | % | % | % |
| Endo, 10, 50 | 4, 2.6 | −3.20 | 1.05 | −1.24 | 49.99 | −193.31 | 13.51 |
| | 6.2, 1.4 | −6.60 | −0.06 | −0.70 | −8.31 | −107.61 | 8.39 |
| Mid, 10, 50 | 4, 2.6 | 5.37 | −7.22 | 3.59 | −60.56 | 23.96 | −6.48 |
| | 6.2, 1.4 | 3.89 | −7.30 | −0.52 | 38.35 | 33.54 | −21.50 |
| Epi, 20, 60 | 4, 2.6 | −2.17 | 6.17 | −2.35 | 10.57 | 169.35 | −7.02 |
| | 6.2, 1.4 | 2.71 | 7.37 | 1.23 | −30.04 | 74.07 | 13.11 |

Figure S6 shows the variations in T wave markers computed from three simulated cases (C316, C352, C532) for a larger range of simulated $[\mathrm{Ca}^{2+}]$ (0.8, 1, 1.2, 1.4, 2, 2.6 and 3.2 mM).

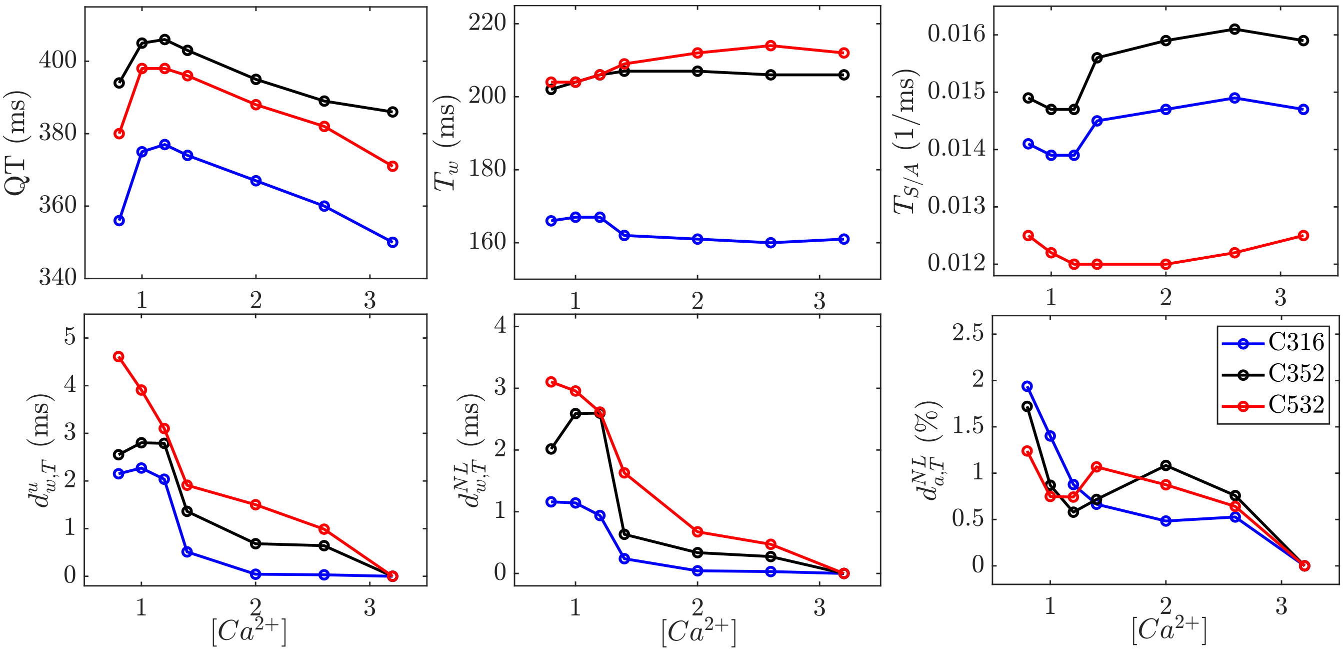


Figure S6: Changes in T wave markers at varying $[\mathrm{Ca}^{2+}]$, from 0.8 to 3.2 mM, for three different simulated cases (C316, C352, C532).
